# Material patterning on substrates by manipulation of fluidic behavior

**DOI:** 10.1093/nsr/nwz034

**Published:** 2019-03-18

**Authors:** Yitan Li, Hao Wang, Henglu Xu, Shiting Wu, Xuemei Li, Jiapeng Yu, Chaoyu Huang, Zeyao Zhang, Hao Sun, Lu Han, Meihui Li, Anyuan Cao, Zhenhai Pan, Yan Li

**Affiliations:** 1 Key Laboratory for the Physics and Chemistry of Nanodevices, Beijing National Laboratory of Molecular Sciences, State Key Laboratory of Rare Earth Materials Chemistry and Applications, College of Chemistry and Molecular Engineering, Peking University, Beijing 100871, China; 2 Academy for Advanced Interdisciplinary Studies, Peking University, Beijing 100871, China; 3 College of Engineering, Peking University, Beijing 100871, China; 4 Electron Microscopy Laboratory, Peking University, Beijing 100871, China; 5 Bruker (Beijing) Scientific Technology Co., Ltd., Beijing 100081, China; 6 Institute of Engineering Thermophysics, Shanghai Jiao Tong University, Shanghai 200240, China

**Keywords:** Marangoni flow, temperature gradient, large-scale material patterning, flexible device, contact line

## Abstract

Patterned materials on substrates are of great importance for a wide variety of applications. In solution-based approaches to material patterning, fluidic flow is inevitable. Here we demonstrate not only the importance of fluidic behavior but also the methodology of engineering the flow pattern to guide the material crystallization and assembly. We show by both experiment and simulation that substrate heating, which is generally used to accelerate evaporation, produces irregular complex vortexes. Instead, a top-heating–bottom-cooling (THBC) set-up offers an inverse temperature gradient and results in a single Marangoni vortex, which is desired for ordered nanomaterial patterning near the contact line. We then realize the fabrication of large-scale patterns of iodide perovskite crystals on different substrates under THBC conditions. We further demonstrate that harnessing the flow behavior is a general strategy with great feasibility to pattern various functional materials ranging from inorganic, organic, hybrid to biological categories on different substrates, presenting great potential for practical applications.

## INTRODUCTION

Patterned functional materials (especially nano- or micro-structured ones) on substrates have been widely applied in biological detection [1], chemical sensing [2], optical imaging [3], optoelectronics [4,5], integrated electronics [6,7] and so on. Solution-based approaches such as spin coating, dip coating, and solution shearing have been intensively investigated for patterning materials on substrates. In these solution approaches, the formation of a thin liquid film is normally necessary [8–14]. Evaporation then happens at the air–liquid interface and substrate heating is often used to accelerate the evaporation. It is obvious that the evaporation affects the local saturation of the solution, which then influences the nucleation and assembly of the solutes (materials). Another essential factor in this process is the mass transfer, which strongly depends on fluid flows.

Different solution processes offer different flow patterns basically attributed to the mechanical forces applied, e.g. the solution shearing process features laminar flow. Therefore, efforts have been made to manipulate the intrinsic flow pattern accordingly. For instance, to overcome the mass transfer limitations of lamellar flow, Bao *et al*. introduced a shearing blade with micropillars to generate a fluid recirculation of the solution [12,13].

Evaporation and heating inevitably bring about temperature differentiation in the solution. Then the Marangoni flow appears spontaneously due to the variation in surface tension, which is temperature dependent [15,16]. This flow offers an additional pathway to harness the fluid flows and mass transfer to realize controllable crystallization and assembly of materials [17].

Here we build a wedge-shaped meniscus without introducing any mechanical forces to study the manipulation of flow behavior and its effect on the material assembly. We find by both experiment and computational fluid dynamics (CFD) simulation that multiple Marangoni vortexes are created under ambient and substrate heating condition but a top-heating–bottom-cooling (THBC) set-up creates a steady Marangoni flow with a single vortex, which is then used to guide the ordered crystallization and assembling of different materials. This shows itself to be a general strategy to obtain ordered patterns of functional materials ranging from inorganic, organic, hybrid, to biological categories (Supplementary Scheme 1). This strategy can be extended to the preparation of large-scale organized material patterns, offering great potential in real applications.

## RESULTS AND DISCUSSION

**Figure 1.**
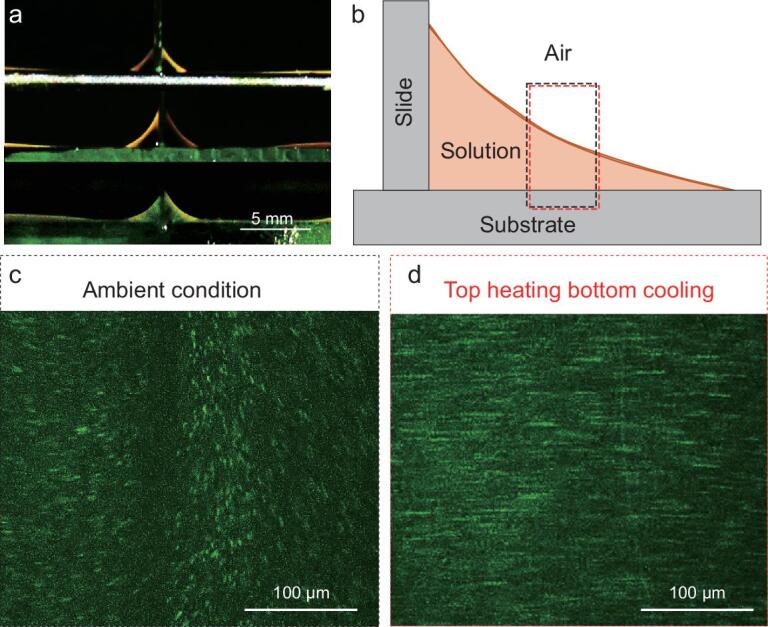
Photo images of the wedge-shaped meniscus with thin liquid films on different substrates: Si/SiO_2_, PET, and glass substrates from top to bottom (a); schematic illustration (b). Photo images of fluorescent particle tracers corresponding to the fluid flow in the liquid wedge under ambient (c) and top-heating–bottom-cooling (THBC) conditions (d).

We created an asymmetric liquid film by using a standing slide on the substrate to generate heterogeneous evaporation [18–21], which may offer additional factors to manipulate the assembly of materials. As shown in Fig. 1a and b, a wedge-shaped meniscus near the standing glass slide with an extended thin liquid film was formed by putting N,N-dimethylformamide (DMF) solution of iodide perovskite [22,23] precursor (CH_3_NH_3_I and PbI_2_) on different substrates of silicon wafer, polyethylene terephthalate (PET), and glass. By selecting proper combinations of solvents, substrates and standing slides, we also succeeded in building such liquid meniscuses of solutions or dispersions of C_60_ [24], 2,4,5-triphenylimidazole (TPI), 9,10-bis(phenylethynyl)anthracene (BPEA) [25], and Ag nanowires. The liquid meniscuses of different solvents including DMF, isopropanol, water, and *m*-xylene are shown in Supplementary Figs 1–4, respectively.

Then, taking a DMF solution of iodide perovskite as an example, we investigated the fluid flows in the liquid wedges under different conditions. We recorded the flow patterns at different heating conditions by using fluorescent polystyrene microspheres in the DMF solution of perovskites as tracers. The videos were taken from the bottom view at the center part of the wedge-shaped meniscus, shown with a dotted rectangle in Fig. 1b. Under ambient conditions, multiple vortexes with unstable fluid flows in the wedge were observed (Fig. 1c and Supplementary Video S1). When a top-heating–bottom-cooling (THBC) action was applied to the system (Fig. 1d and Supplementary Video S3), a uniform and stable single vortex appeared in the liquid wedge. We also recorded the flow patterns under THBC at the tip (Supplementary Video S2) and the end (Supplementary Video S4) of the liquid wedge. The results clearly show that there is only one vortex within the liquid wedge. This regular flow should be preferable for the preparation of ordered material patterns.

**Figure 2.**
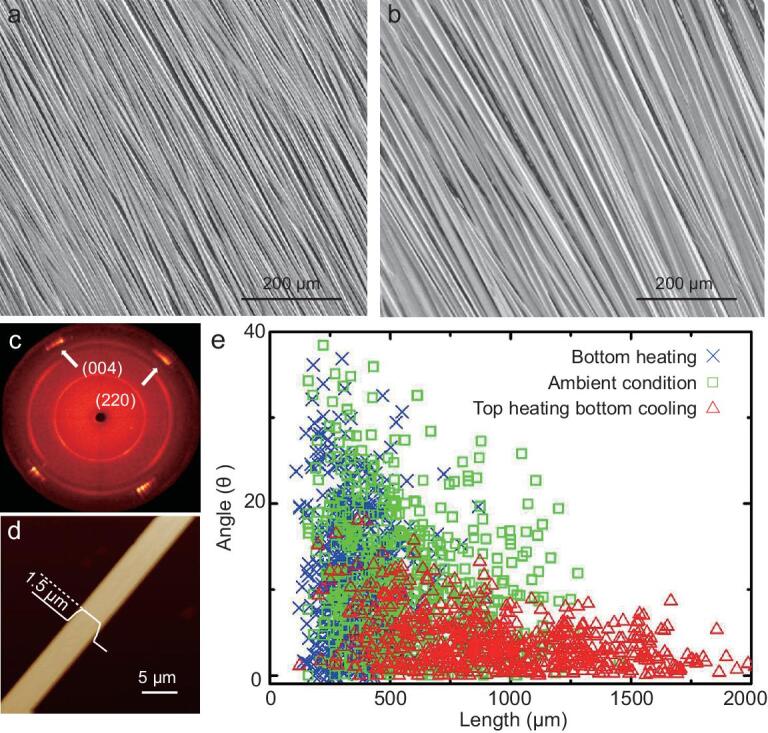
SEM image of aligned CH_3_NH_3_PbI_3_ on Si/SiO_2_ (a) and PET (b) with corresponding 2D XRD pattern (c) and AFM image (d). (e) Statistics of the length and alignment of CH_3_NH_3_PbI_3_ wires grown under different heating conditions.

Well-aligned CH_3_NH_3_PbI_3_ arrays were indeed fabricated under suitable THBC conditions (Supplementary Fig. 5a) on Si/SiO_2_, PET, and glass substrates as shown in Fig. 2a, b, and Supplementary Fig. 6a. The X-ray diffraction patterns shown in Supplementary Fig. 6b indicate that the products were tetragonal CH_3_NH_3_PbI_3_. An atomic force microscopy (AFM) image shows that each feature has a smooth surface, revealing a single crystal structure (Fig. 2d). The high-resolution transmission electron microscopy (HRTEM) image in Supplementary Fig. 6c exhibits fringes perpendicular to the longitudinal direction of the crystal with the inter-distance of ∼3.2 Å, which are in good accordance with the reported data of (0 0 4) planes. The corresponding selected area electron diffraction (SAED) pattern is also in good agreement. In the 2D wide angle X-ray diffraction (WAXD) image, four groups of diffraction spots appear in pairs; these were assigned to the (0 0 4) and (2 2 0) planes of CH_3_NH_3_PbI_3_ crystals, respectively (Fig. 2c). The WAXD image also reveals that the perovskite crystals are aligned and grow along the (0 0 1) direction or *c*-axis. These characterizations all indicate that our method can be used to grow oriented crystal arrays with high crystallinity. In contrast, if the solution is left under ambient conditions at all, the resultant crystals are in poor alignment with dendritic structures. Traditional bottom heating resulted in discontinuous crystals with poor alignment (Supplementary Fig. 7).

Then we tried to quantitatively compare the effects of heating conditions on the length and alignment of CH_3_NH_3_PbI_3_ crystals. We defined the axis perpendicular to the contact line of the liquid wedge as the standard direction and measured the angle between the CH_3_NH_3_PbI_3_ wires and this axis to depict the alignment. We measured 1855 CH_3_NH_3_PbI_3_ wires in the SEM mages and show the statistical results in Fig. 2e and Supplementary Fig. 8. The CH_3_NH_3_PbI_3_ wires grown under THBC conditions present the largest lengths and the narrowest distribution of angles, exhibiting the best alignment and continuities (Table S1 in the supplementary data online).

**Figure 3.**
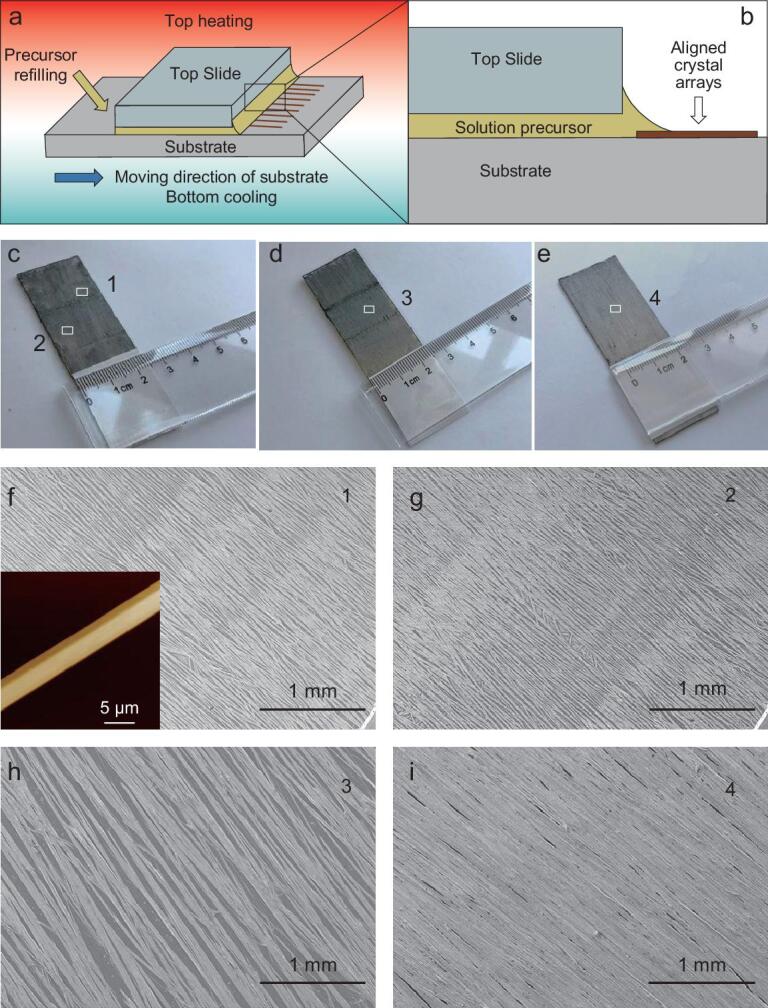
The preparation of large-scale patterns. (a, b) Schematic illustrations of the slipping-substrate set-up under top-heating–bottom-cooling with zoomed solution meniscus area where the aligned crystal arrays grew. Photographs of centimeter-scale CH_3_NH_3_PbI_3_ crystal arrays grown on glass with different thicknesses of the liquid films and concentrations of the precursors: the concentration (wt) of precursor in the samples is 30% (c), 30% (d), and 45% (e) and the thickness of the liquid films is 50 μm (c), 150 μm (d), and 150 μm (e), respectively. (f–i) SEM images of CH_3_NH_3_PbI_3_ crystal arrays corresponding to the regions numbered in (c–e).

The scale of the pattered materials is limited by the size of the wedge-shaped meniscus. In order to prepare large-area patterns of functional materials, we designed a slipping-substrate set-up as illustrated in Fig. 3a and b. Under THBC conditions, CH_3_NH_3_PbI_3_ wires formed at the margin of the wedge area of the solution. By slipping the substrate, CH_3_NH_3_PbI_3_ crystals grew continuously. As shown in Fig. 3c–e, CH_3_NH_3_PbI_3_ were patterned uniformly over centimeters of the substrates with good continuity and alignment (Fig. 3f–i). The patterned materials were well crystalized with a regular morphology and smooth surface (Fig. 3f, inset). The morphology, crystallinity, and density of the crystals can be easily adjusted by changing the thickness of the liquid film, the concentrations of the solution, and the THBC conditions. When increasing the thickness of the liquid film, the size of the CH_3_NH_3_PbI_3_ crystals increased correspondingly (Fig. 3f–h). A higher concentration of precursors produced denser crystal arrays (Fig. 3h and i). The above results show that with steady control of the movement of the slipping substrate, the solution supply and, more importantly, the THBC conditions, large-scale well-aligned crystalline CH_3_NH_3_PbI_3_ arrays can be produced. Such kinds of large-scale material arrays should be of great potential in real applications.

The above results show the importance of flow behavior in patterning materials. Understanding the mechanism of the formation of ordered fluid flow is essential for the rational design of the material patterning process. As shown in Supplementary Video S5, the unstable flow in ambient conditions spontaneously changes to a stable single vortex when a heat flux is applied from top, indicating the essential influence of temperature gradient on the fluid flow. Under THBC conditions, the surface of the solution presents a higher temperature than the bottom, while the situation is reversed under ambient conditions due to the surface evaporation of the solvent. Thus, it is reasonable to assume that Marangoni flow is generated in this system from the variation in surface tension due to the temperature gradient, similar to the situation near the bubble surface in a boiling system [15].

Then, we simulated the flow behavior in such a system with sophisticated computational fluid dynamics (CFD) modeling, which has been validated in a series of liquid-wedge evaporation experiments on various substrates [26]. A 2D description of the fluid wedge was adopted. Sophisticated numerical treatments [27] were imposed on the liquid–gas interface to fully take into account the cooling effect as well as the concentration increase of the solute due to the solvent evaporation (Supplementary Fig. 9). Note that, due to the very fine meshing in the thin film region near the contact line, a double-precision calculation and strict convergence criteria are necessary to avoid a fake low-rate diffusion in the fine mesh area. The surface tension along the meniscus varied with local temperature and local solute concentration. Marangoni flow was therefore induced.

**Figure 4.**
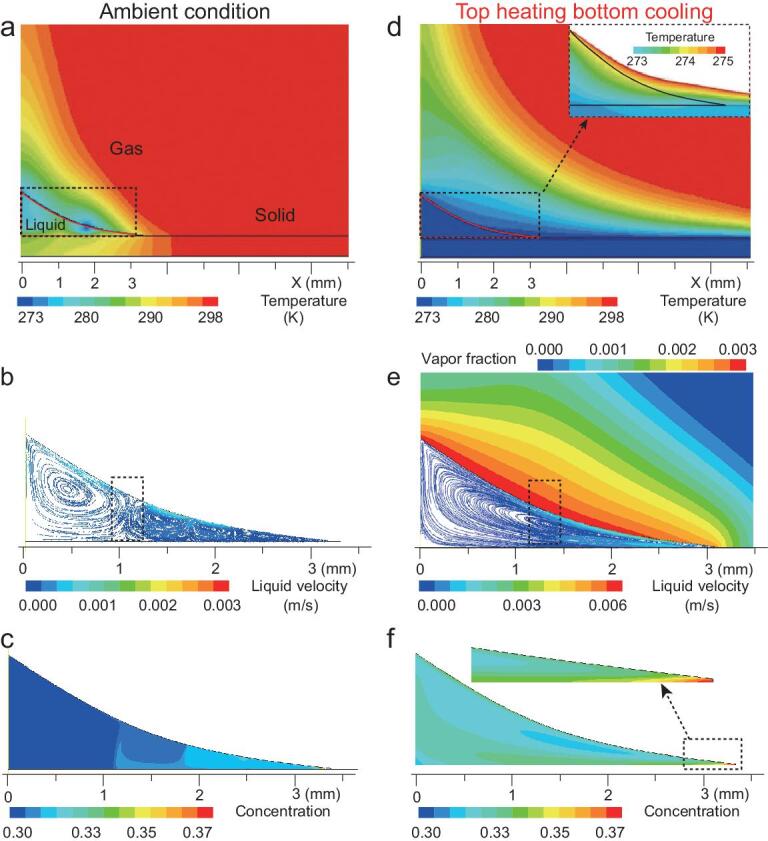
The computational fluid dynamics simulation of a liquid wedge under ambient conditions and the top-heating–bottom-cooling (THBC) set-up. The temperature field of the modeling system under ambient conditions (a) with corresponding liquid flow field (b) and solute concentration (c) of the liquid wedge. The temperature field of the modeling system under THBC set-up and zoomed meniscus area (inset) (d) with corresponding liquid flow field (e) and solute concentration (f) of the liquid wedge.

When leaving the system under ambient conditions, a disordered distribution of temperature (Fig. 4a) and solute (Fig. 4c) in the liquid wedge was observed. This was caused by the complicated vortexes in the solution wedge (Fig. 4b). These vortexes increased the instabilities of the mass transfer process and dispersed the solution with high concentration back into the wedge zone. When conventional bottom heating was applied, the fluid flow in the meniscus showed similar patterns with faster fluid flows (Supplementary Fig. 10). However, under THBC conditions, the temperature was monotonously dropping from bulk to the thin film corner (Fig. 4d). The temperature difference along the meniscus is around 2 K and the Marangoni number was calculated to be ∼18 380, which is greater than some reported data [28,29]. The temperature gradient resulted in a surface tension gradient [30] and consequently the Marangoni flow was formed with a single vortex occupying the liquid wedge (Fig. 4e). This single Marangoni vortex carried the solutes to the tip of the liquid wedge and the highest concentration of solutes was therefore located there. Therefore, the single vortex with uniform and stable fluid flows in the liquid wedge under THBC conditions is important for the crystal growth in two ways. Firstly, the flow provides guidance to the directionality of the crystal growth since the flow is always perpendicular to the crystallization front. Secondly, because of the co-effect of the steady Marangoni flow and solvent evaporation, the highest concentration of solutes is always located at the tip of the thin film corner. All these simulated results are in good agreement with our experimental results.

**Figure 5.**
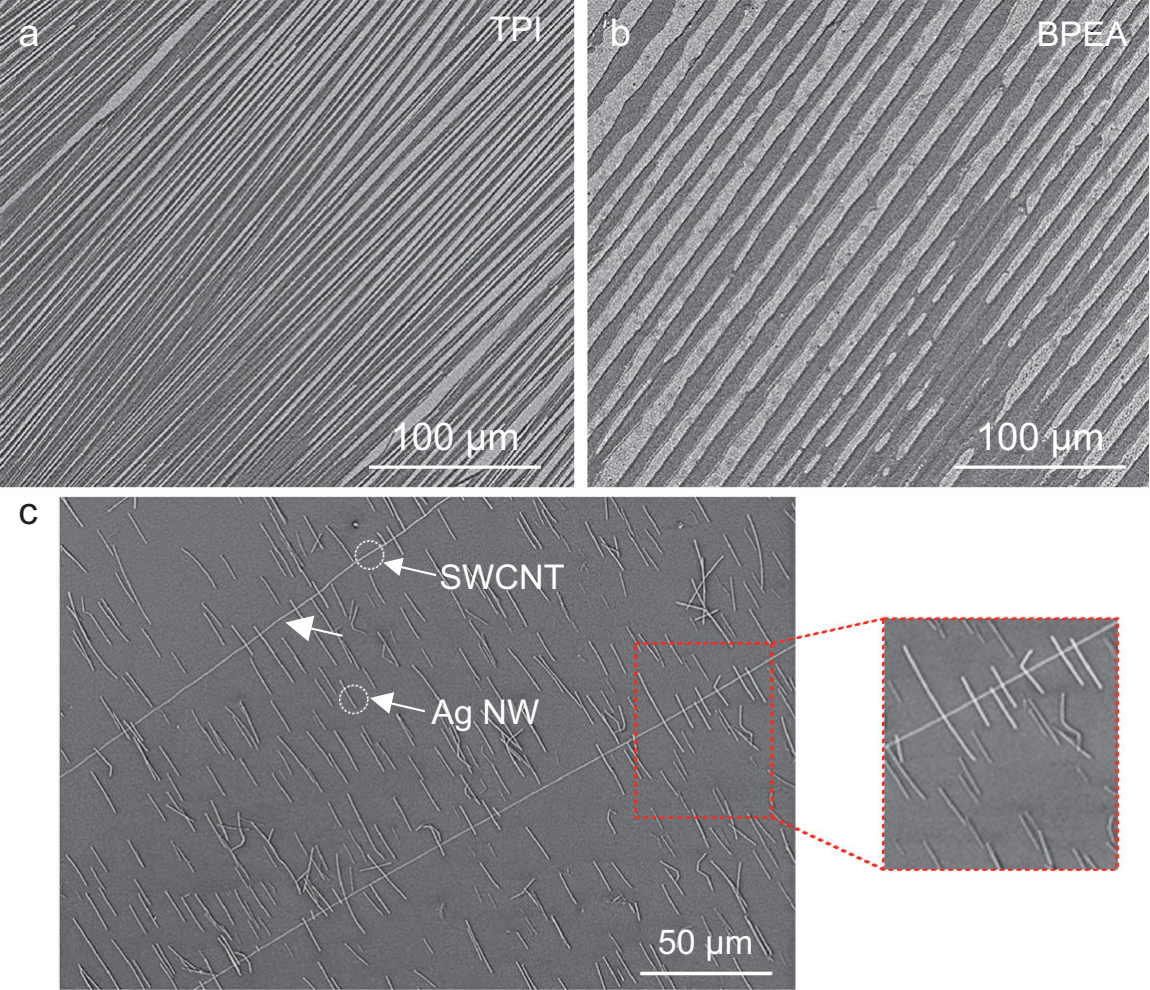
Formation of TPI, BPEA and organized Ag nanowires arrays. SEM images of aligned TPI grown from DMF (a) and BPEA grown from *m*-xylene (b). (c) SEM images of aligned Ag nanowires on Si/SiO_2_ with aligned single-walled carbon nanotubes.

Our strategy shows great tolerance and feasibility and exhibits its validity in a series of material-solvent systems. The top heating and bottom cooling synergistically modulate the evaporating rate of the solvent. Every material has an applicable growth window for aligned patterns depending on the properties of both the material and the solvent, as shown in Supplementary Figs 5, 11a and b. DMF has higher latent heat and boiling point than *m*-xylene; as a result, ordered patterns formed in the zones with higher bottom temperature and higher densities of top heating flux for the DMF solution (Supplementary Fig. 11a and b). Besides CH_3_NH_3_PbI_3_ arrays, TPI (Fig. 5a), BPEA (Fig. 5b and Supplementary Fig. 11c) and C_60_ crystal arrays (Supplementary Fig. 11d) were also fabricated by our strategy.

The THBC set-up offers a reversed Marangoni flow and the liquid velocity can be adjusted over quite a broad range. Therefore, besides the molecular solutes, the nanometer- to micrometer-sized dispersants may also be manipulated. We chose a dispersion of Ag nanowires in DMF as an example to explore the transfer and deposition of such meso-scaled dispersants under the THBC set-up (Supplementary Fig. 12). When the reversed Marangoni flow is strong enough, the Ag nanowires may be combed by the fluid flow to form aligned structures perpendicular to the contact line of the liquid with the substrate. We thus obtained aligned Ag nanowires on both Si/SiO_2_ and PET substrates. But weak fluid flow resulted in random Ag nanowires. When we performed this procedure on a silicon wafer with pre-grown horizontally aligned ultralong single-walled carbon nanotubes (SWNTs) and made the contact line of the liquid parallel to the orientation of SWNTs, the Ag nanowires were deposited on the substrate and formed cross structures with the tubes, as shown in Fig. 5c. When the evaporating rate was too high, the Ag nanowires were pinned at the contact line, which is very similar to the ‘coffee ring’ formed in the normal evaporation process of a droplet [20,21,31–33].

**Figure 6.**
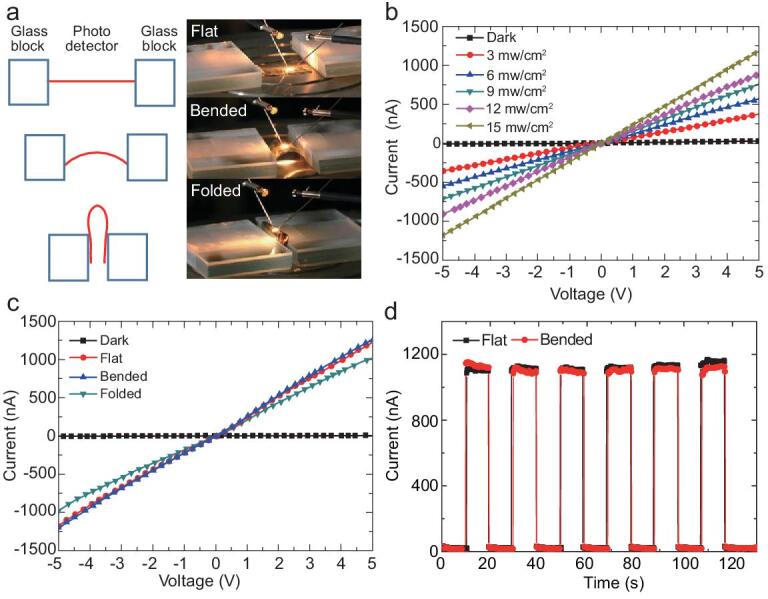
Formation and performance of perovskite-array-based photodetectors. Schematic illustration with corresponding photo images of a perovskite-based photodetector tested under different deformations (flat, bent and folded) (a) and corresponding *I*–*V* (b, c), *I*–*T* (d) curves. The light intensity is ∼15 mW/cm^2^.

Lead halide perovskites have long-range electron–hole diffusion lengths [34,35] as well as high balanced hole and electron mobility and are therefore excellent materials for photovoltaic devices [36]. The THBC set-up enables us to directly fabricate aligned millimeter-scale crystals on flexible substrates. These arrays of crystals present fewer grain boundaries and hence lower trap density, longer lifetime of photo-generated carriers and smaller recombination rate in devices. We applied CH_3_NH_3_PbI_3_ arrays prepared on PET substrates immediately to fabricate long-channel photodetectors by evaporating Au electrodes with Cr buffer layers. The device showed excellent flexibility and sensitivity to incident light with different intensities (Fig. 6a and b). Bending did not cause any significant change in the photo response performances (Fig. 6c and d), indicating that our devices are tolerant to large bending deformations. Moreover, when the channel length of the photodetector was extended to 1.5 mm, no obvious deterioration of photo response performance was observed (Supplementary Fig. 13c) owing to the good crystallinity of the perovskite arrays. Photodetectors based on perovskite arrays obtained without THBC showed poorer performance, but still better than the spin-coated perovskite film (Supplementary Fig. 13d and e). The performance of the devices is comparable with reported ones though no annealing was applied to the aligned crystals.

## CONCLUSIONS

Fluidic behavior plays a key role in solution-based material assembling processes relying on its contribution to the mass transfer. Manipulating the temperature gradient is a feasible approach to harness fluid flows. Applying a proper flux of heat from the evaporating side of the liquid film can build a temperature gradient inverse to the conventional substrate-heating mode. Thus a single Marangoni vortex is created within the liquid film, bringing the solutes to the contact-line region of the solution meniscus and facilitating the ordered assembly and patterning of nanomaterials. This method is efficient and feasible with validity for a variety of materials, solvents, and substrates. It is also applicable for patterning materials over large areas. We believe that it is a general strategy to prepare patterns of different functional materials on various substrates for further applications.

## METHODS

### Materials

#### Solutions of materials or precursors

The 0.3077 g CH_3_NH_3_I (99.9%, Dyesol, Australia) and 0.8923 g PbI_2_ (99.9%) (molar ratio 1:1) were dissolved into 2.8 g N,N-dimethylformamide (DMF) to get a precursor solution of 30 wt% for CH_3_NH_3_PbI_3_. A precursor solution of 45 wt% was prepared similarly. 2,4,5-triphenylimidazole (TPI) was dissolved into DMF with a concentration of 5 mg/mL. 9,10-Bis(phenylethynyl)anthracene (BPEA) was dissolved into DMF and *m*-xylene, respectively, with a concentration of 1 mg/mL. C_60_ was dissolved into *m*-xylene with a concentration of 1 mg/mL. Ag nanowires were synthesized following the method reported previously [37] and dispersed in DMF with a concentration of 2 mg/mL.

#### Substrates

All substrates were ultrasonicated with ethanol and water for 5 min respectively and rinsed with water. Then Si/SiO_2_ and glass substrates were treated in piranha solution (H_2_SO_4_:H_2_O_2_ 7:3, v/v) at 80°C for 40 min and PET films were treated with air plasma for 5 min. The ultralong single-walled carbon nanotubes were grown by chemical vapor deposition (CVD) on Si/SiO_2_ substrate following the conditions in our previous paper [38].

#### Preparation of material patterns

The precursor solution was dropped on the pretreated substrate and a standing slide was placed vertically on the substrate. The top-heating–bottom-cooling set-up was realized via the following method. Two infrared lamps (275 W and 100 W) were applied on top. The distance between the 275 W infrared lamp and the substrate was adjusted from 20 cm to 50 cm, corresponding to the top heat flux from ∼275 to 100 W/m^2^. The distance between the 100 W infrared lamp and substrate was adjusted between 20 cm and 30 cm, corresponding to the heat flux from ∼77 W/m^2^ to 44 W/m^2^. The heat flux applied by the infrared lamps was calibrated. A semiconductor cooling plate was placed under the substrate with a temperature ranging from −10°C to 30°C. For the bottom-heating process, the substrate was placed on a hot plate with the temperature ranging from 30°C to 70°C.

For the growth of large-scaled aligned CH_3_NH_3_PbI_3_ crystal arrays, a glass brick (2.5 cm × 7.5 cm × 2.5 mm) was used instead of the standing slide on the substrate to sandwich the precursor solution of CH_3_NH_3_PbI_3_. A stepper motor was applied to move the substrates horizontally at a speed of 20 μm/s, releasing the precursor solution to grow aligned crystal arrays of CH_3_NH_3_PbI_3_ under the top-heating–bottom-cooling set-up.

#### Characterizations

The CH_3_NH_3_PbI_3_ microribbons grown on substrates were directly characterized with a cold field emission scanning electron microscope (Hitachi S4800, operated at 2.0 kV, 10 μA) and an X-ray diffractometer (D/MAX-PC 2500, Rigaku, with Cu Ka radiation at λ = 0.154 nm). A Bruker D8 Discover diffractometer with a general area detector diffraction system (GADDS) as a 2D detector was applied for 2D wide angle X-ray diffraction (WAXD) measurements. The calibration was done with silicon powder and silver benzoate. The TEM specimen was prepared by rubbing the grid with the microribbon arrays. The high-resolution TEM characterization was carried out on a FEI Tecnai G2 T20 microscope. A Dimension Icon SPM (Bruker, Santa Barbara, CA, USA) was used to perform the AFM topographic measurements of the CH_3_NH_3_PbI_3_ arrays. The photo response performance of the devices was studied under ambient conditions using a tungsten lamp with a power density ranging from 0 to 15 mW/cm^2^.

#### Numerical modeling

The modeling is partially based on Wang *et al*.’s previous numerical work on an evaporating liquid wedge in a groove [27]. A 2D description of the wedge system is adopted. The simulation domains, i.e. liquid, solid, and gas, are discretized as shown in Supplementary Fig. 9. The meniscus is sustained between the horizontal substrate and the vertical symmetric surface. Its geometry is set up based on the optical observation. The ambient temperature and vapor fraction are fixed at the outer boundary of the gas domain. The gas domain size is, as shown in Supplementary Fig. 9a, 3.3 times the meniscus length and 10 times the meniscus height. The domain is discretized. The thin film near the contact line with thickness less than10 μm has been truncated as shown in Supplementary Fig. 9c. The coefficients of the surface tension to the temperature and concentration are listed in Table S2.

#### Problem description

Solute mass diffusion and advection in the liquid have been included in the model. At the liquid–gas interface the evaporative cooling is accounted for. Vapor transport in the gas domain is coupled to the evaporation since the vapor is produced at the interface and then transferred into the gas domain. The change of solution concentration due to the solvent evaporation on the interface is also accounted for. The volume change by evaporation is significantly slower than the transport processes; therefore a quasi-steady assumption for the meniscus shape and wedge volume during the evaporation process was appropriate; this has been proved in the literature [39–41]. Under this assumption, the heat and mass transport behavior depends only on the instantaneous liquid geometry and environment conditions. Governing equations and numerical treatments are described below.

#### Evaporation and vapor transport at the meniscus

A higher interfacial temperature, *T*_lv_, brings more energetic liquid molecules to cross the interface into vapor. A higher vapor pressure, *P*_v_lv_, brings more vapor molecules to condense. Using the kinetics-based Hertz-Knudsen-Schrage equation we can evaluate the net evaporated mass flux, *m*^″^_net_, at the interface [42–44]:

(1) \begin{equation*} {m''_{{\rm{net}}}} = \frac{{2\bar{\sigma }}}{{2 - \bar{\sigma }}}{\left( {\frac{M}{{2\pi R}}} \right)^{1/2}}\left( {\frac{{{p_{{\rm{v}}\_{\rm{equ}}}}{T_{\rm{lv}}}}}{{T_{{rm{lv}}}^{1/2}}} - \frac{{{p_{{\rm{v}}\_{\rm{lv}}}}}}{{T_{{\rm{v}}\_{\rm{lv}}}^{1/2}}}} \right) \end{equation*}

where *P*_v_equ_(*T*_lv_) is the equilibrium vapor pressure that could be approximated as the saturation pressure, *P*_sat_(*T*_lv_), if the effects of the disjoining and the capillary pressure are neglected. By the Clausius–Clapeyron equation,

(2) \begin{eqnarray*} {p_{{\rm{v}}\_{\rm{equ}}}}({T_{{\rm{lv}}}}) \approx {p_{{\rm{sat}}}}({T_{{\rm{lv}}}})\nonumber\\ = {p_{{\rm{sat}}\_{\rm{ref}}}}\exp \left( {\frac{{M{h_{{\rm{fg}}}}}}{R}\left( {\frac{1}{{{T_{{\rm{sat}}\_{\rm{ref}}}}}} - \frac{1}{{T_{{\rm{lv}}}}}}\right)}\right). \end{eqnarray*}

Assuming that the vapor and liquid have about the same temperature at the interface, which could be all right for low heat flux cases, then

(3) \begin{eqnarray*} && {m''_{{\rm{net}}}} &=& \frac{{2\bar{\sigma }}}{{2 - \bar{\sigma }}}{\left( {\frac{{\bar{M}}}{{2\pi \bar{R}}}} \right)^{1/2}} \nonumber\\ && \frac{1}{{T_{{\rm{lv}}}^{1/2}}}({p_{{\rm{v}}\_{\rm{equ}}}}({T_{{\rm{lv}}}}) - {p_{{\rm{v}}\_{\rm{lv}}}}). \end{eqnarray*}

*P*_v_lv_ is related to the vapor transport in the gas domain, which is described by

(4) \begin{equation*}\frac{{\partial {C_{\rm{v}}}}}{{\partial t}} + \vec{v} \cdot \nabla {C_{\rm{v}}} = \nabla \cdot (D \cdot \nabla {C_{\rm{v}}}).\end{equation*}

Then from the gas domain point of view, the mass flux on the gas side of the interface due to vapor transport is

(5) \begin{equation*} {m''_{\rm{v}}} = M \cdot J = {\left. {M \cdot \left( { - D\frac{{\partial {C_{\rm{v}}}}}{{\partial n}} + {v_{\rm{n}}}{C_{\rm{v}}}} \right)} \right|_{{\rm{lv}}}}.\end{equation*}

The first term on the right represents the diffusion. The second represents the advection component. *v*_n_ ensures that the net mass transport of air is zero since air does not cross the interface as vapor. At the interface, the evaporating mass flux must be equal to the vapor transport mass flux such that *m*^″^_net_ = *m*^″^_v_, which provides the link between the evaporation and the vapor transport. For details refer to [27]. Besides the evaporation calculation, the solute transport in the liquid due to diffusion as well as advection is included.

#### Numerical treatment

The pressure-based finite-volume scheme [45] implemented in FLUENT was employed for the numerical calculation. The fluid properties are shown in Table S2 [46–48]. All the nomenclature is listed in Table S3.

The surface tension along the meniscus varies with local temperature and local solute concentration. Marangoni flow is therefore induced. The coefficients of surface tension to the temperature as well as concentration have been listed in Table S3. The local surface tension at temperature *T* and solute concentration *C*_sol_ is determined by

(6) \begin{equation*}\gamma = {\gamma _0} + \frac{{d\gamma }}{{d{C_{{\rm{sol}}}}}}({C_{{\rm{sol}}}} - {C_{{\rm{sol}},0}}) + \frac{{d\gamma }}{{dT}}(T - {T_0}).\end{equation*}

The reference γ0 is 37.12 mN/m at *T*_0_ being room temperature and *C*_sol,0_ being 20%.

The evaporative cooling effect due to latent heat is achieved by employing energy sources at the mesh cells at the interface. Mass sources due to phase change are also added. Adjustments are also made on the sensible and other scalars related to the mass change. The solute concentration keeps increasing when the solvent keeps evaporating. In the simulation we first obtain the temperature and flow field and then investigate the solute concentration. The results of the solute concentration distribution in this paper and this material are all 12 s after the solvent evaporation starts. The initial concentration is set to be 0.3.

For the simulated top-heating–bottom-cooling (THBC) set-up, a heat flux of 275 W/m^2^ is imposed on the liquid surface and the solid upper surface, while the bottom surface of the solid is cooled with a fluid temperature of 273.15 K and the convection heat transfer coefficient is approximated as 100 W/m^2^ K. For the simulated traditional bottom-heating set-up, the bottom temperature was 313.15 K while the ambient temperature was 298.15 K. Due to the very fine meshing near the thin film region, a double-precision calculation and strict convergence criteria are necessary; otherwise a fake low-rate diffusion will occur in the fine mesh area.

## Supplementary Material

nwz034_Supplemental_Files
