# Supplementary material for: Material patterning on substrates by manipulation of fluidic behavior
Source: Natl Sci Rev. 2019 Mar 18;6(4):758–66. doi: 10.1093/nsr/nwz034 (PMC8291501; doi:10.1093/nsr/nwz034)
Supplement: nwz034_Supplemental_Files [file nwz034_supplemental_files.zip › Supplementary_Information-revised.docx]

**Supplementary information**

**Material Patterning on Substrates by Manipulation of Fluidic Behavior**

*Yitan Li^1,2^, Hao Wang^3,^*, Henglu Xu^1^, Shiting Wu^3^, Xuemei Li^4^, Jiapeng Yu^3^, Chaoyu Huang^3^, Zeyao Zhang^1^, Hao Sun^5^, Lu Han^1^, Meihui Li^1^, Anyuan Cao^3^, Zhenhai Pan^6^, Yan Li^1,2,^**

^1^Key Laboratory for the Physics and Chemistry of Nanodevices, Beijing National Laboratory of Molecular Sciences, State Key Laboratory of Rare Earth Materials Chemistry and Applications, College of Chemistry and Molecular Engineering, Peking University, Beijing 100871, China.

^2^Academy for Advanced Interdisciplinary Studies, Peking University, Beijing 100871, China.

^3^College of Engineering, Peking University, Beijing 100871, China

^4^Electron Microscopy Laboratory, Peking University, Beijing 100871, China

^5^Bruker (Beijing) Scientific Technology Co., Ltd., Beijing 100081, China

^6^Institute of Engineering Thermophysics, Shanghai Jiao Tong University, Shanghai 200240, China

Correspondence to: [yanli@pku.edu.cn](mailto:yanli@pku.edu.cn) & [hwang@coe.pku.edu.cn](mailto:hwang@coe.pku.edu.cn)

**Content**

**Scheme 1** Cartoon schematic illustration of traditional bottom-heating setup and top-heating-bottom-cooling setup for the growth of large-scale crystal arrays················1

**Supplementary Figure 1.** The wetting performance of DMF with different vertical slides and horizontal substrates·································································2

**Supplementary Figure 2.** The wetting performance of isopropanol with different vertical slides and horizontal substrates·······················································3

**Supplementary Figure 3.** The wetting performance of water with different vertical slides and horizontal substrates································································4

**Supplementary Figure 4.** The wetting performance of m-xylene with different vertical slides and horizontal substrates·······························································5

**Supplementary Figure 5.** THBC conditions for the patterning of perovskite and TPI from DMF as well as C_60_ and BPEA from m-xylene, respectively·························6

**Supplementary Figure 6.** Characterizations of CH_3_NH_3_PbI_3_ arrays······················7

**Supplementary Figure 7.** SEM images of CH_3_NH_3_PbI_3_ microribbon arrays grown at different conditions·····························································8

**Supplementary Figure 8.** Growing conditions of CH_3_NH_3_PbI_3_ perovskite arrays······9

**Supplementary Figure 9.** Schematic diagram of the open liquid wedge················10

**Supplementary Figure 10.** The liquid flow field and solute concentration of the meniscus under bottom-heating setup··················································11

**Supplementary Figure 11.** The formation of BPEA and C_60_ crystal patterns·········12

**Supplementary Figure 12.** SEM images of Ag nanowires patterned on different substrates under different conditions and the THBC conditions for patterning Ag nanowires on silicon substrates·······························································13

**Supplementary Figure 13.** Photovoltaic characteristics of CH_3_NH_3_PbI_3_ prepared at different conditions·············································································14

**Table S1.** Average length and Parallelity of the CH_3_NH_3_PbI_3_ Crystals Grown under Different Conditions············································································15

**Table S2.** Properties of fluids·································································16

**Table S3.** Nomenclature·······································································17

**Supplementary Video S1** Flow patterns in the middle part of the liquid wedge under ambient condition.

**Supplementary Video S2** Flow patterns at the tip of the liquid wedge under top-heating-bottom-cooling setup.

**Supplementary Video S3** Flow patterns in the middle part of the liquid wedge under top-heating-bottom-cooling setup.

**Supplementary Video S4** Flow patterns at the end of the liquid wedge under top-heating-bottom-cooling setup.

**Supplementary Video S5** The changes of the flow patterns in the middle part of the liquid wedge when a heat flux was applied from top.

*
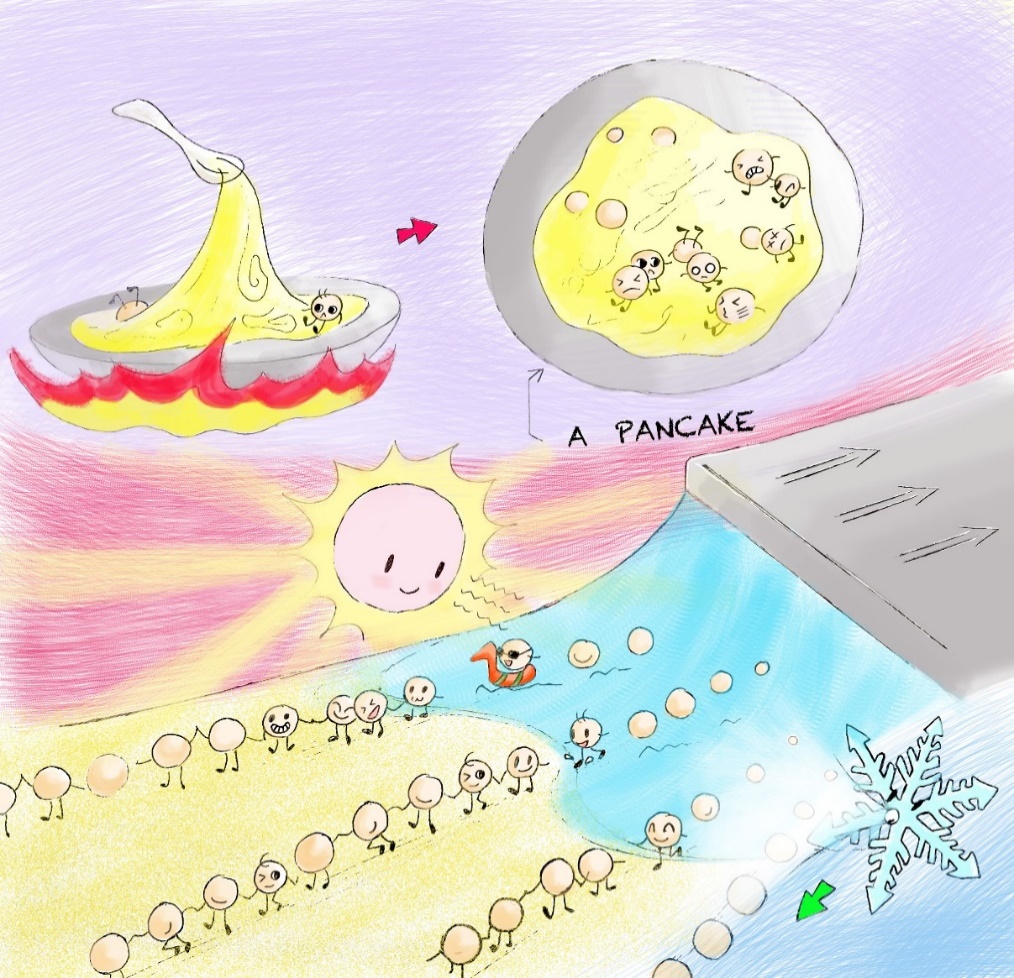
*

**Scheme 1** Cartoon schematic illustration of traditional bottom-heating setup (up) and top-heating-bottom-cooling setup for the growth of large-scale crystal arrays (down).


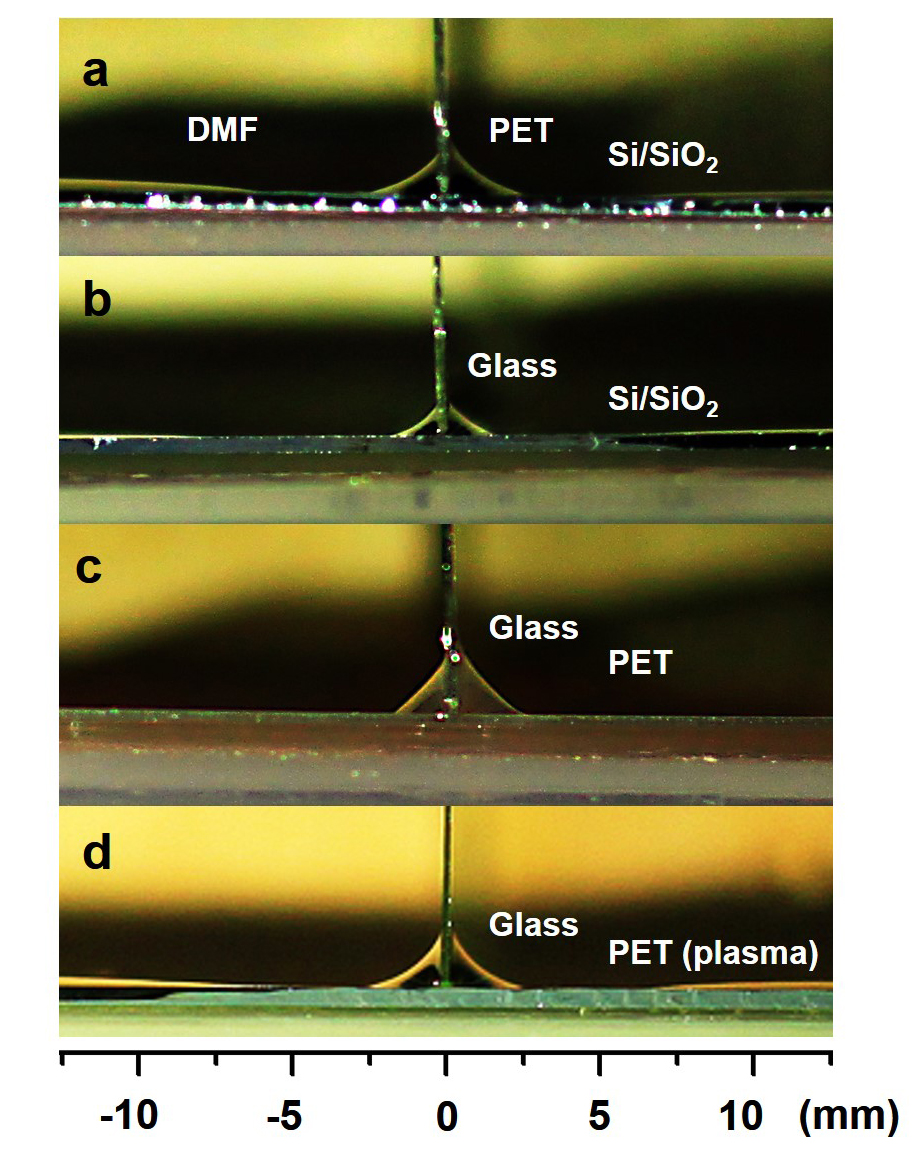


**Supplementary Figure 1.** The wetting performance of DMF with different vertical slides and horizontal substrates.


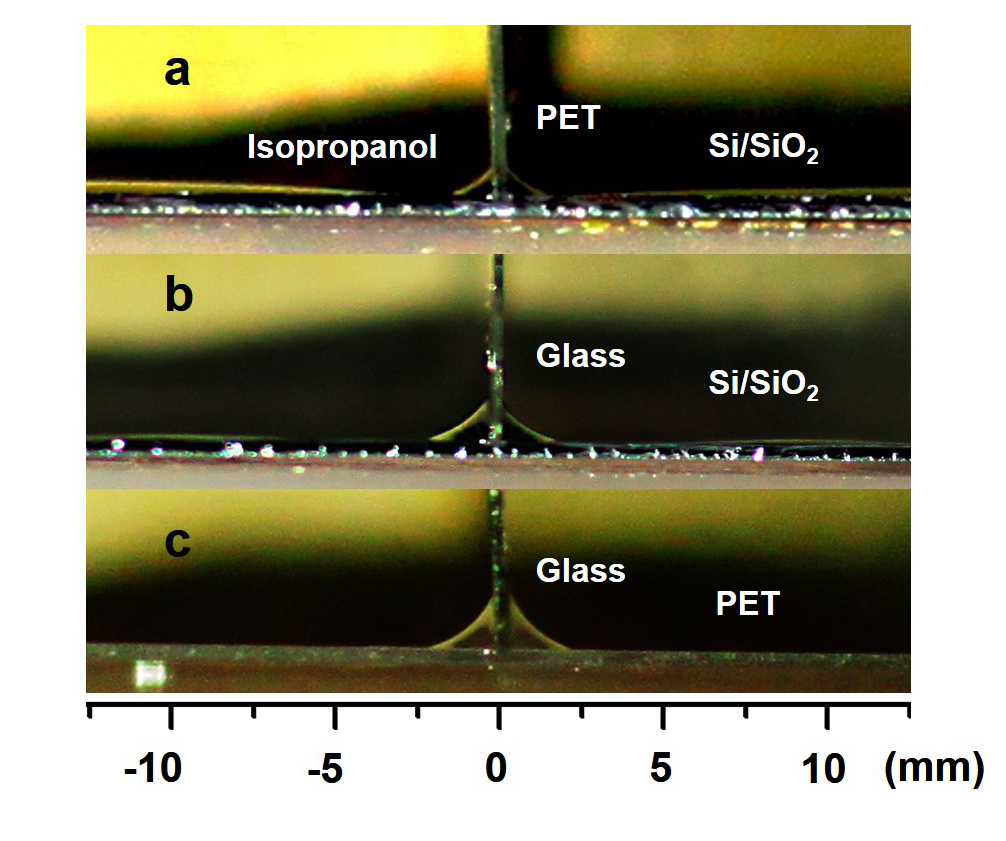


**Supplementary Figure 2.** The wetting performance of isopropanol with different vertical slides and horizontal substrates.


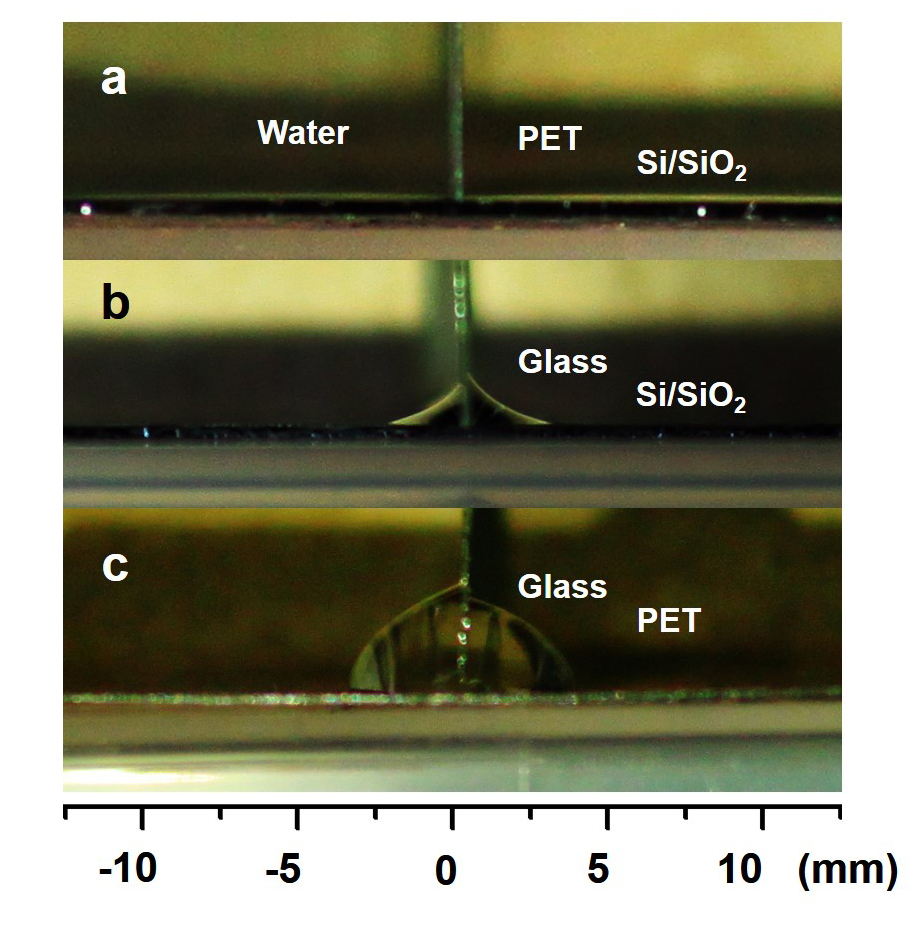


**Supplementary Figure 3.** The wetting performance of water with different vertical slides and horizontal substrates.


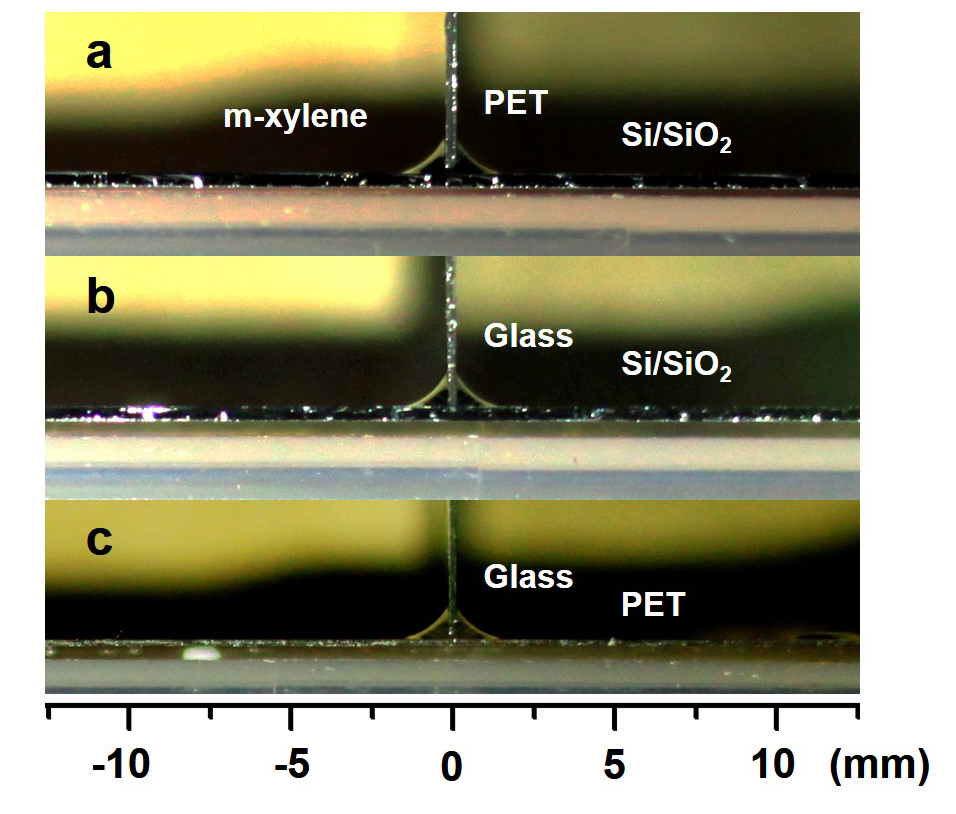


**Supplementary Figure 4.** The wetting performance of m-xylene with different vertical slides and horizontal substrates.


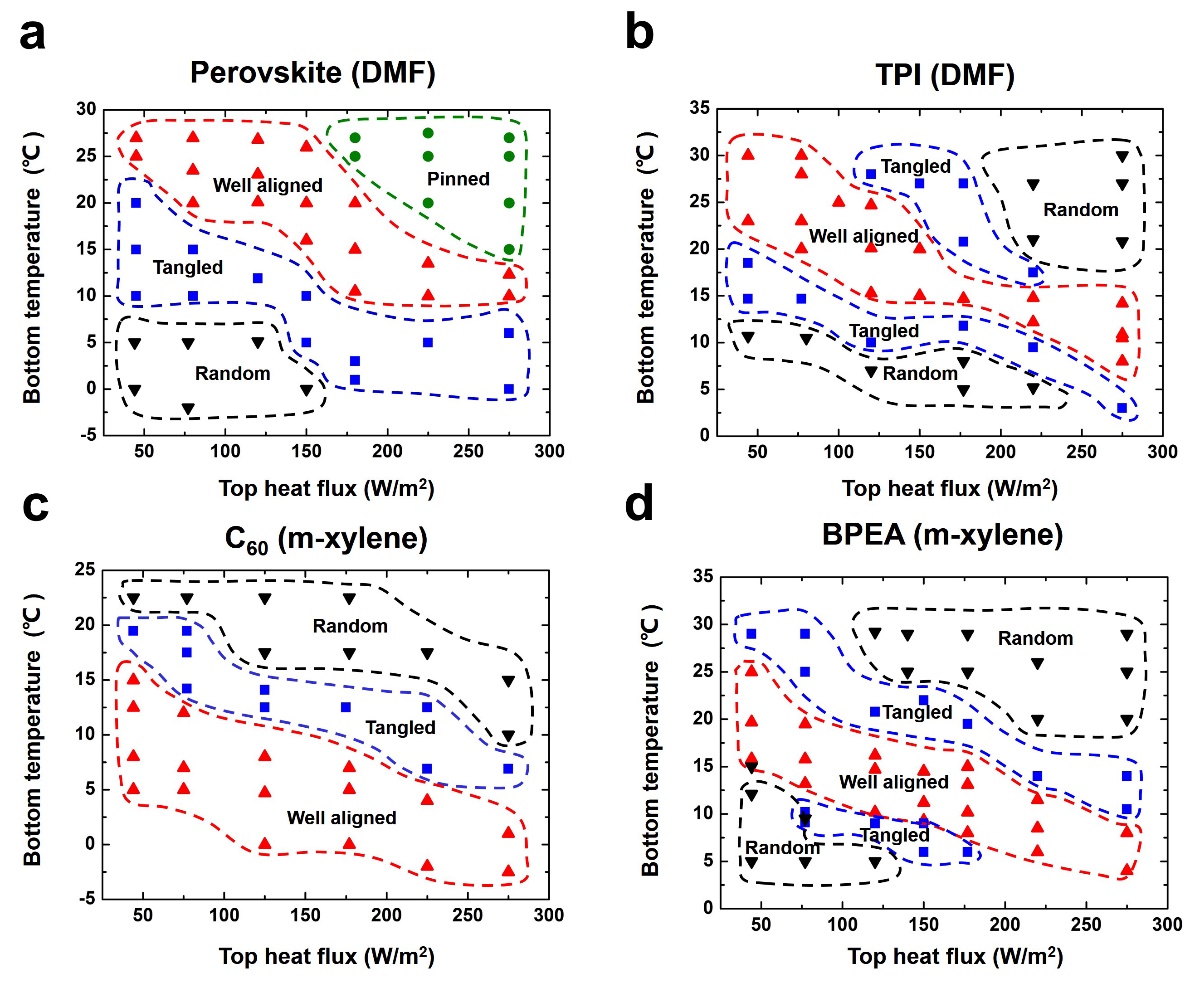


**Supplementary Figure 5. THBC conditions for the patterning of perovskite (a) and TPI (b) from DMF as well as C_60_ (c) and BPEA (d) from m-xylene, respectively.**


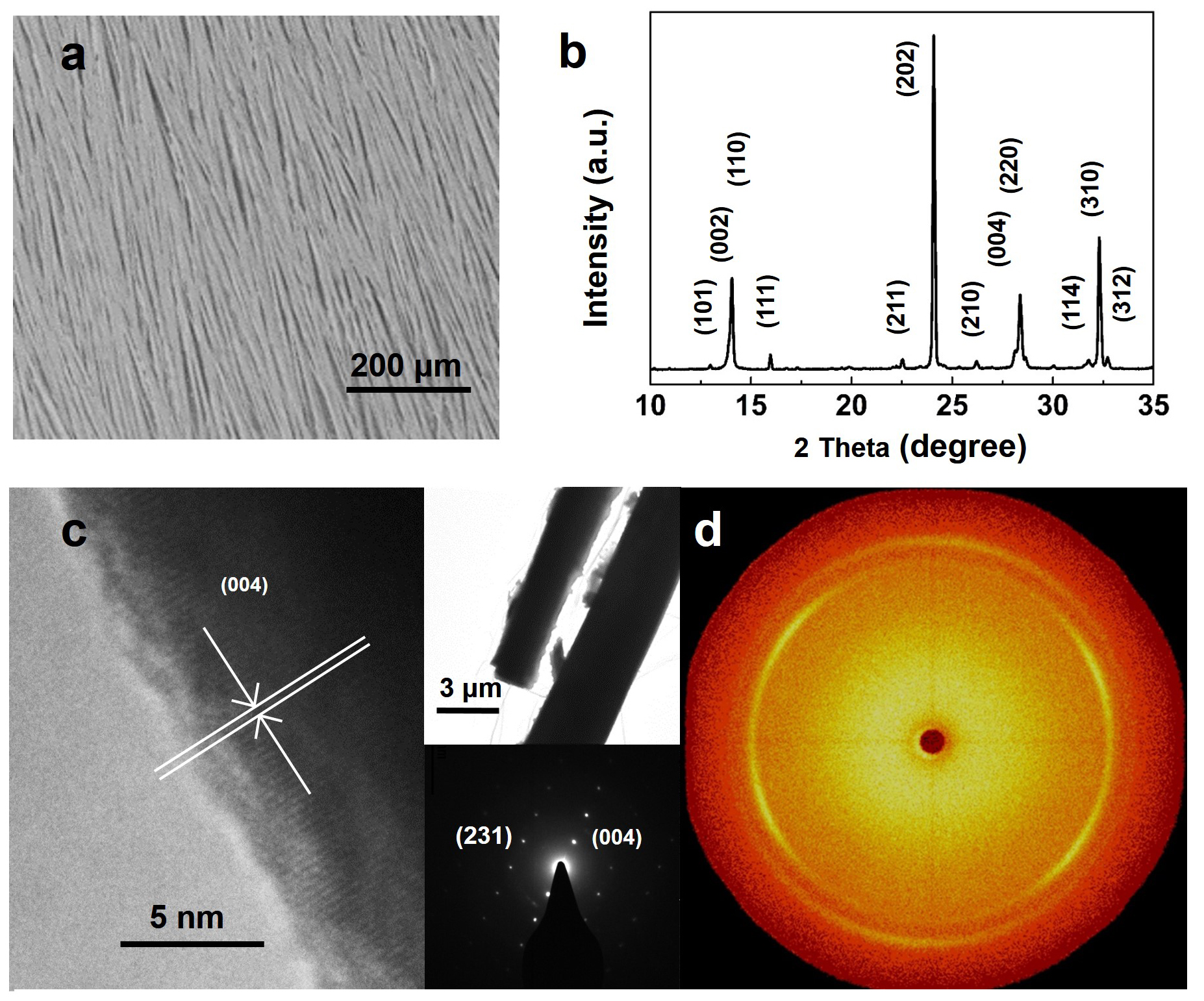


**Supplementary Figure 6. Characterizations of CH_3_NH_3_PbI_3_ arrays. a**, SEM images of CH_3_NH_3_PbI_3_ arrays on glass. **b,** One-dimensional X-ray diffraction patterns. **c,** High resolution transmission electron microscopy (HRTEM) image with corresponding SAED pattern inserted. **d,** Two-dimensional wide angle X-ray diffraction (WAXD) image of PET film.


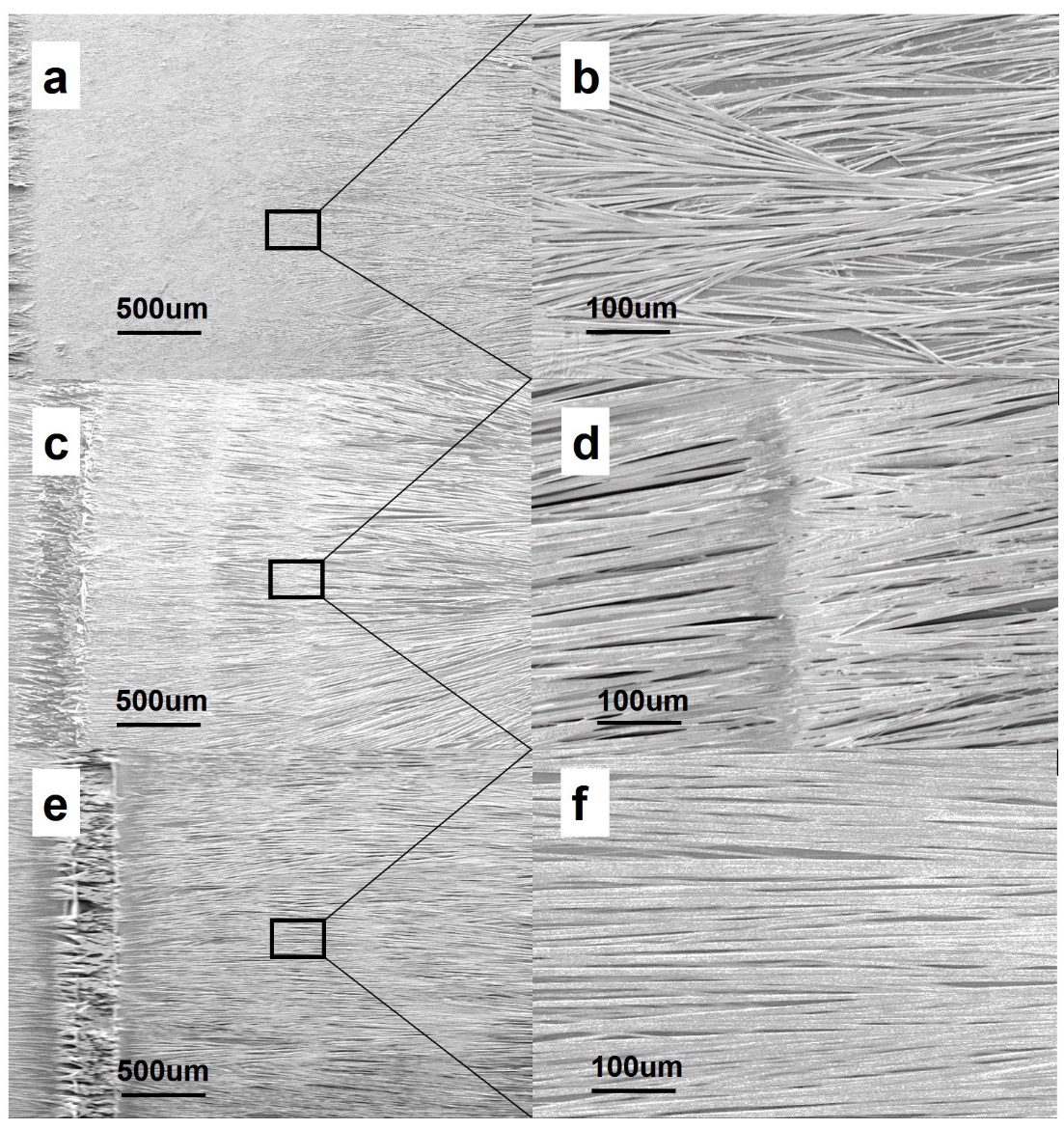


**Supplementary Figure 7. SEM images of CH_3_NH_3_PbI_3_ microribbon arrays grown at different conditions.** **a & b**, ambient condition; **c & d**, bottom heating; **e & f**, top-heating-bottom-cooling.


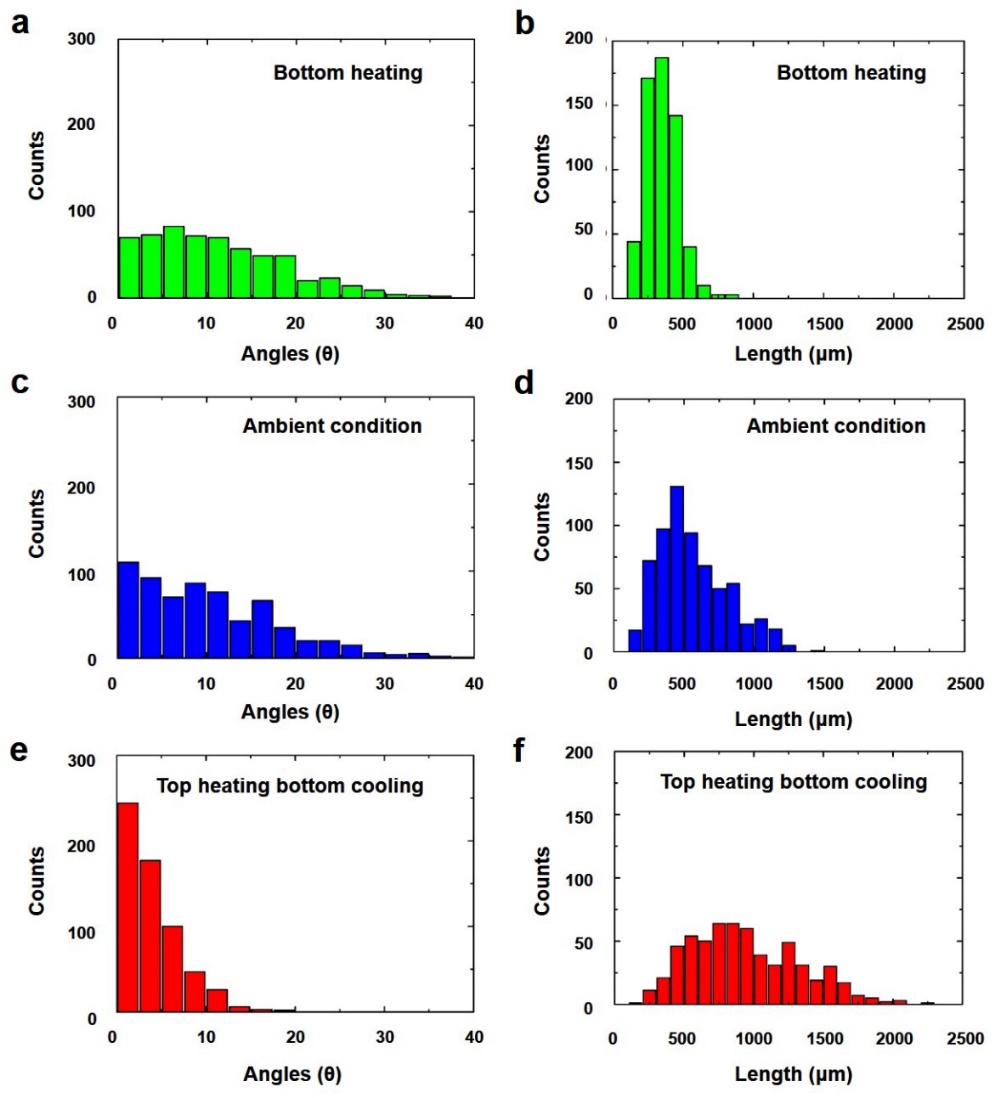


**Supplementary Figure 8. Growing conditions of CH_3_NH_3_PbI_3_ perovskite arrays.** Statistic results of lengths and angles of CH_3_NH_3_PbI_3_ micro ribbons grown under bottom heating (a & b), ambient (c & d) and top-heating-bottom-cooling (THBC) (e & f) conditions.


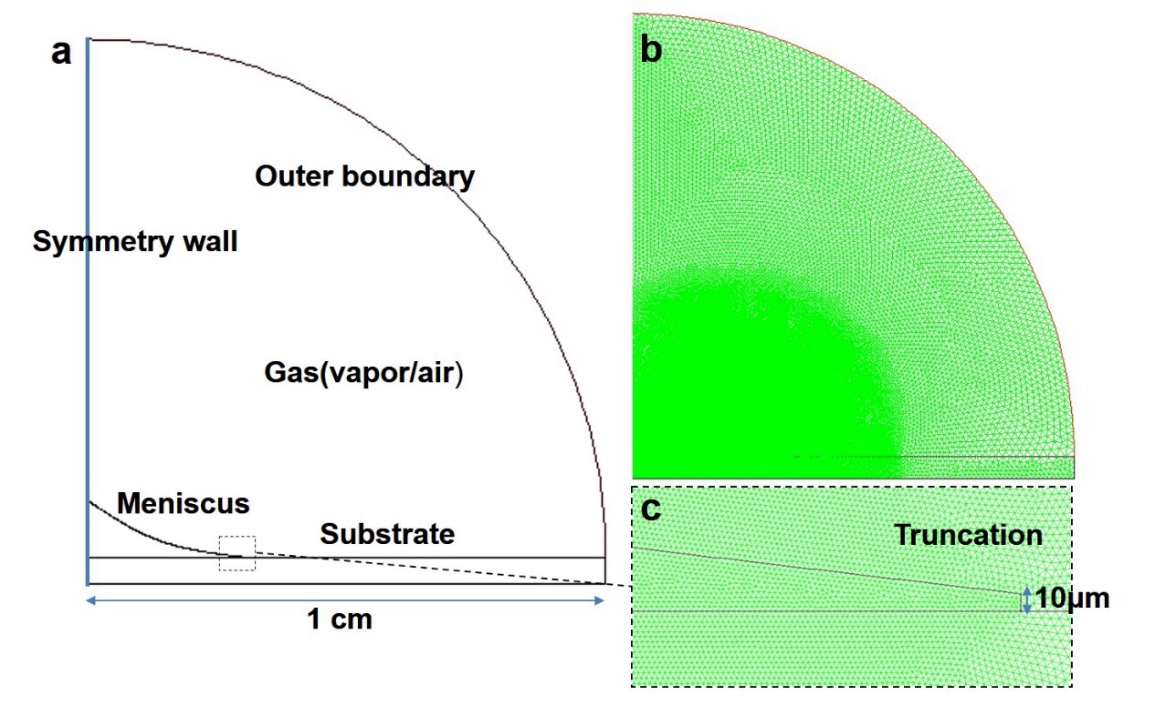


**Supplementary Figure 9. Schematic diagram of the open liquid wedge** with an evaporating meniscus (a), mesh setup for the system (b) and details of the mesh in the vicinity of the thin film corner (c).


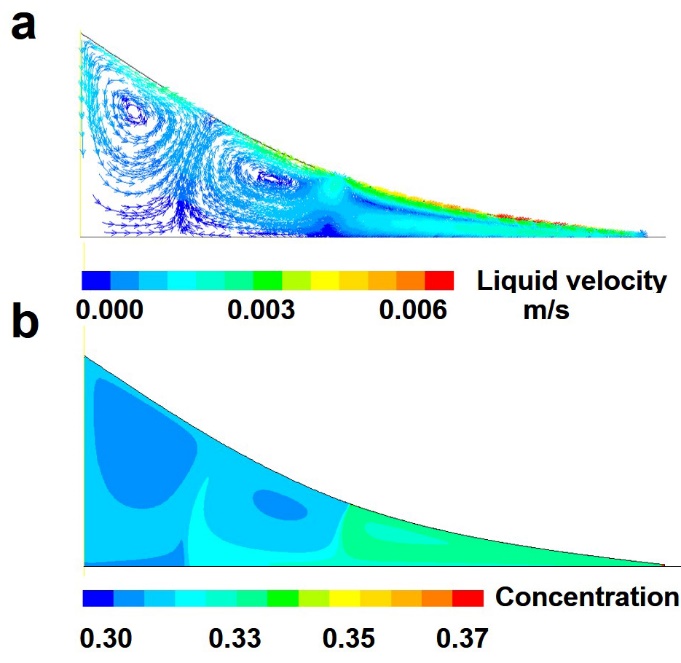


**Supplementary Figure 10. The liquid flow field (a) and solute concentration (b) of the meniscus under bottom-heating setup.**


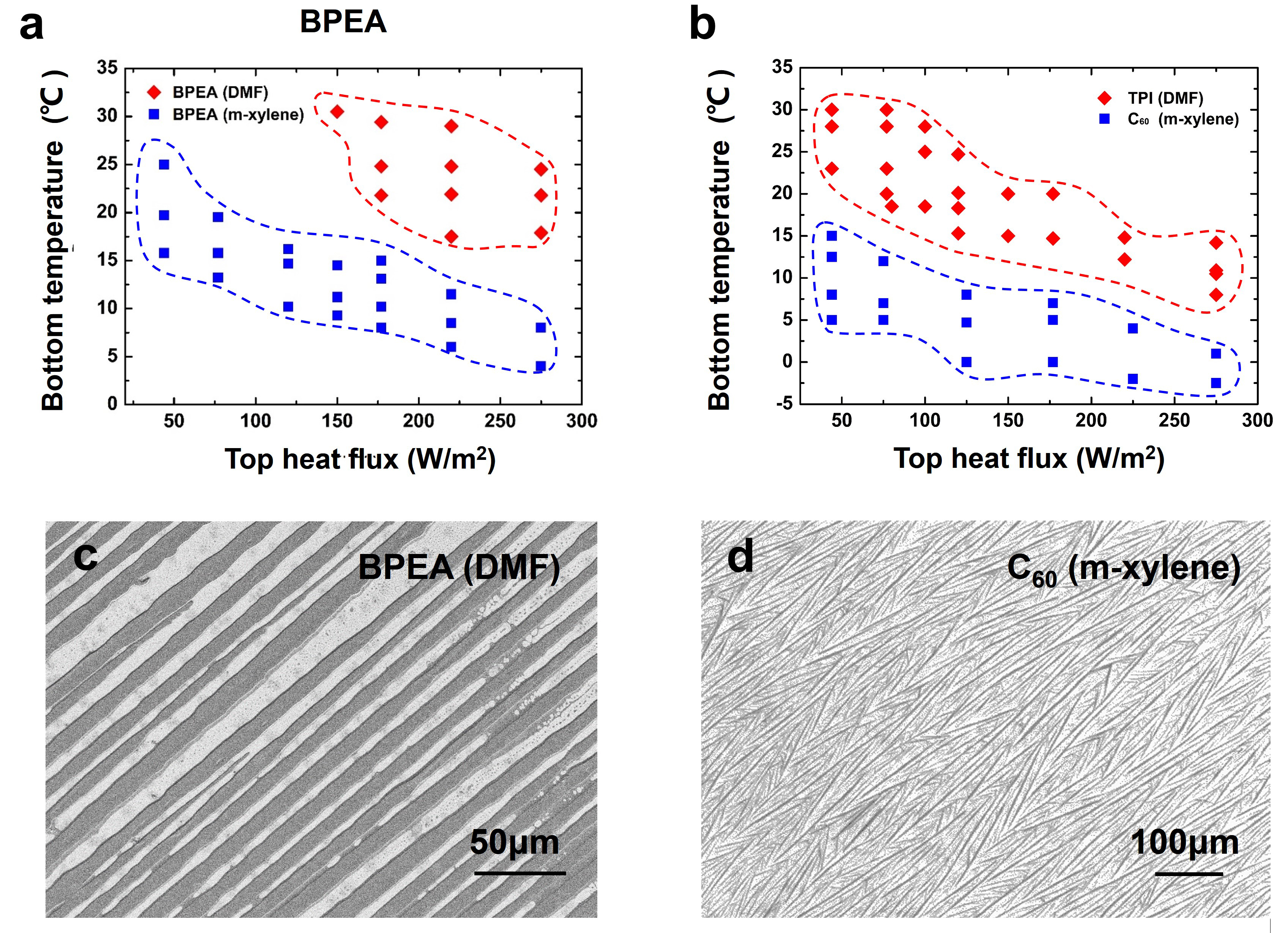


**Supplementary Figure 11. The formation of BPEA and C_60_ crystal patterns. a & b**, THBC conditions for patterning BPEA from m-xylene and DMF (a), C_60_ from m-xylene and TPI from DMF (b). **c** **& d**, SEM images of aligned BPEA grown from DMF (c) and C_60_ from m-xylene (d).

**
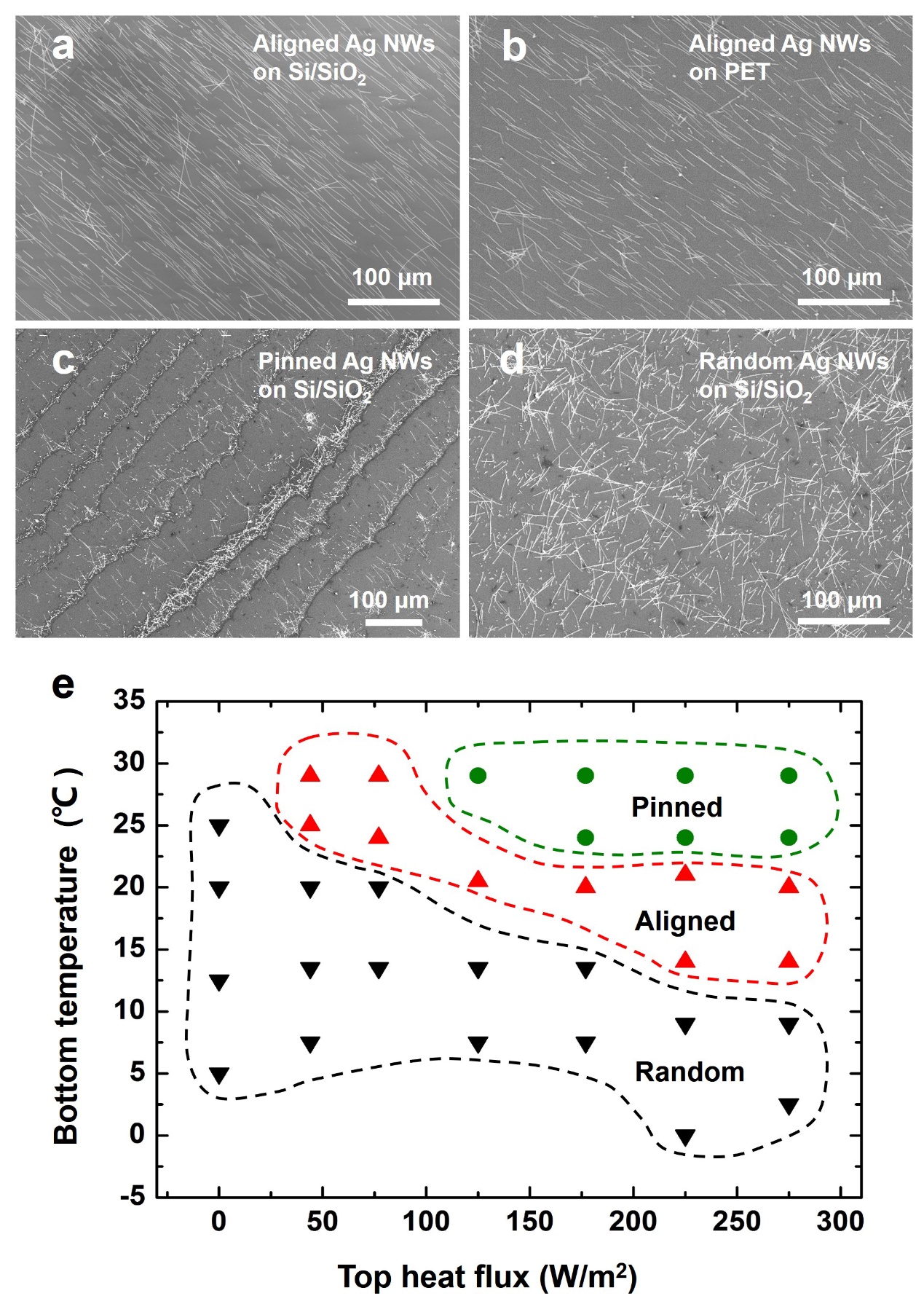
**

**Supplementary Figure 12. SEM images of Ag nanowires patterned on different substrates under different conditions (a-d) and the THBC conditions for patterning Ag nanowires on silicon substrates (e).**


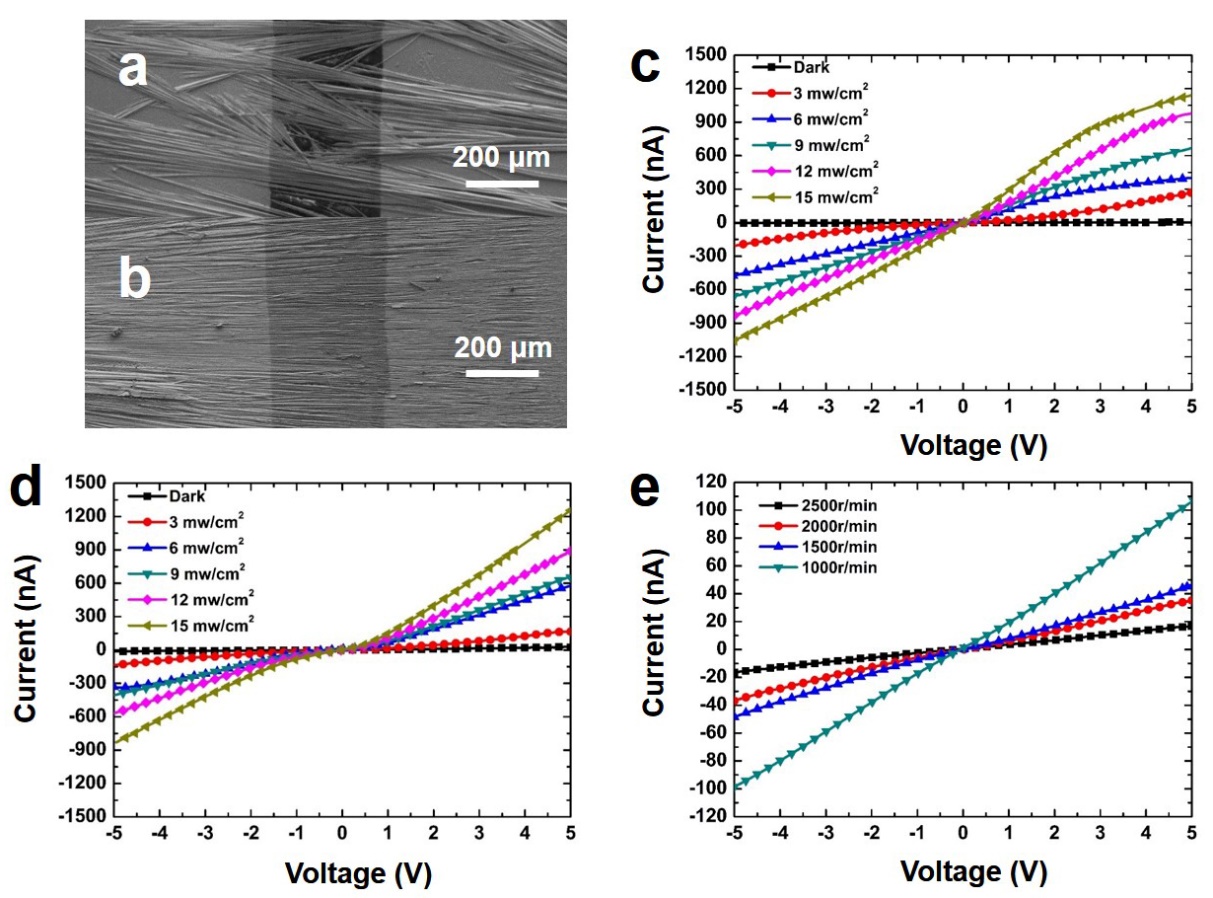


**Supplementary Figure 13. Photovoltaic characteristics of CH_3_NH_3_PbI_3_ prepared at different conditions.** **a & b**, SEM images of perovskite photodetectors fabricated with perovskite crystal arrays grown under ambient condition (a) and under top-heating-bottom-cooling (THBC) setup (b), respectively (scale bars are 200 μm). **c & d**, the photo currents under different light power for perovskite arrays grown at THBC (c) and ambient (d) conditions, the channel length is 1.5 mm. **e**, Photoresponse performance of random perovskite prepared by spin coating.

**Table S1. Average length and Parallelity of the CH_3_NH_3_PbI_3_ Crystals Grown under Different Conditions**

|  | mean value | | root mean square  (RMS) value |
| --- | --- | --- | --- |
| bottom heating | length/μm | 352.4 | 370.8 |
|  | angle/° | 11.54 | 13.68 |
| ambient condition | length/μm | 562.0 | 614.7 |
|  | angle/° | 10.54 | 13.42 |
| top-heating-bottom-cooling | length/μm | 938.7 | 1016.4 |
|  | angle/° | 4.00 | 5.13 |

**Table S2. Properties of fluids**

| Properties | DMF | Air |  |
| --- | --- | --- | --- |
| Density (kg/m^3^) | 999.19 at 298.15 K | Calculated |  |
| Thermal conductivity (W/m K) | 0.186 | 0.0242 |  |
| Thermal capacity (J/ kg K ) | 2054 | 1006.43 |  |
| Viscosity (kg/m s) | 8.123×10^-4^ | 1.789×10^-5^ |  |
| Vapor molecular weight (kg/mol) | 0.073 | 0.029 |  |
| Diffusion coefficient (m^2^/s) | Vapor in air 9× 10^−6^ at 298.15 K | – | |
| Diffusion coefficient (m^2^/s) | Solute in liquid 5× 10^−10^ at 298.15 K  2.54×10^-5^ at 293.65 K | – | |
| Surface tension coefficient of temperature d*γ*/d*T* (N/m K) | -1.83×10^-4^ | – |  |
| Surface tension coefficient of temperature d*γ*/d*C*_sol_ (N/m %) | 10.8×10^-4^ | – |  |
|  |  |  |  |
| Latent heat (J/ kg) | 6.341×10^5^ | – |  |

**Table S3. Nomenclature**

| Variable | Description | Variable | Description |
| --- | --- | --- | --- |
| *C* | vapor molar conc. (mol/m^3^) | *p* | pressure (N/m^2^) |
| *c_p_* | heat capacity (J/kg K) | *R* | universal gas constant (J/mol K) |
| *D* | diffusion coefficient in air (m^2^ /s) | *T* | temperature (K) |
| *h_fg_* | latent heat of evaporation (J/kg) | *V* | fluid velocity (m/s) |
| *k* | thermal conductivity (W/m K) | *q* | heat flux (W/m^2^) |
| *l* | length (m) | *μ* | dynamic viscosity (N s/m^2^) |
| *M* | molecular weight (kg/mol) | *ρ* | density (kg/m^3^) |
| *m’’_net_* | mass flux (kg/m^2^ s) | *γ* | surface tension (N/m) |
| Subscript | Description | Subscript | Description |
| *atm* | atmospheric pressure | *l* | liquid |
| *g* | gas (vapor/air mixture) | *lv* | liquid-gas interface |
| *air* | air | *ref* | reference |
| *v* | vapor | *sat* | saturated |
